# RETIRING AND CAREGIVING IN THE AGE OF STUDENT LOANS: THE IMPACT OF STUDENT DEBT ON RETIREMENT AND LONGEVITY PLANNING

**DOI:** 10.1093/geroni/igz038.163

**Published:** 2019-11-08

**Authors:** Samantha Brady, Julie Miller, Alexa Balmuth, Lisa D'Ambrosio, Joseph Coughlin

**Affiliations:** MIT AgeLab, Cambridge, Massachusetts, United States

## Abstract

Saving for retirement and the ability to provide care for a loved one can be dramatically affected by student loan debt. Currently, approximately 44 million people of all ages in the United States carry the weight of over 1.4 trillion dollars of student loan debt. Student loan borrowers of all ages may experience lower financial preparedness for retirement as well as decreased ability to provide care for family members, including aging parents. While older adults hold a relatively small proportion of student loans, they are the fastest growing subset of student loan borrowers and have disproportionately high rates of student loan defaults. As a result of their defaults, the Social Security retirement benefits of Americans ages 65 and older experienced a 500% increase in offsets over the last decade. This presentation will spotlight an MIT AgeLab mixed methods study about how student loan borrowers between the ages of 51 and 75 experience student loans within family systems and perceive and prioritize longevity planning in light of their student loans. Data collected for this study include focus groups and a large national survey. Preliminary findings suggest that for older borrowers, student loans are generally one of several financial constraints that can inform spending and saving decisions. For most, student loan payments are regarded as stunting overall retirement savings while the minority regard the two separately. Older borrowers also tend to have increased financial and familial responsibilities, including caring for aging parents, that compete for borrowers’ limited financial and temporal resources.

